# Salivary Genomics, Transcriptomics and Proteomics: The Emerging Concept of the Oral Ecosystem and their Use in the Early Diagnosis of Cancer and other Diseases

**DOI:** 10.2174/138920208783884900

**Published:** 2008-03

**Authors:** T.K Fábián, P Fejérdy, P Csermely

**Affiliations:** 1 Clinic of Prosthetic Dentistry, Semmelweis University, Faculty of Dentistry, Budapest, Hungary; 2 Institute of Medical Chemistry, Semmelweis University, Budapest, Hungary

**Keywords:** Saliva, genome, transcriptome, proteome, systemic diseases, oral diseases, screening, early diagnosis.

## Abstract

There is an increasingly growing interest world-wide for the genomics, transcriptomics and proteomics of saliva and the oral cavity, since they provide a non-invasive source of unprecedently rich genetic information. The complexity of oral systems biology goes much beyond the human genome, transcriptome and proteome revealed by oral mucosal cells, gingival crevicular fluid, and saliva, and includes the complexity of the oral microbiota, the symbiotic assembly of bacterial, fungal and other microbial flora in the oral cavity. In our review we summarize the recent information on oral genomics, transcriptomics and proteomics, of both human and microbial origin. We also give an introduction and practical advice on sample collection, handling and storage for analysis. Finally, we show the usefulness of salivary and oral genomics in early diagnosis of cancer, as well as in uncovering other systemic diseases, infections and oral disorders. We close the review by highlighting a number of possible exploratory pathways in this emerging, hot research field.

## INTRODUCTION

**Fig. (1):**
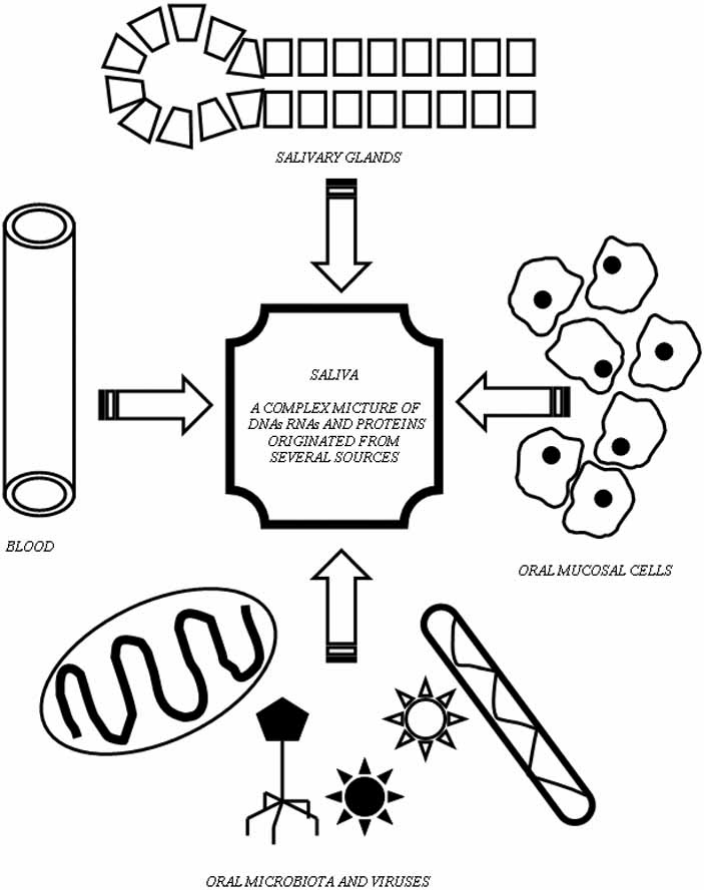
Complexity of salivary genome, transcriptome and proteome.

The genomics, transcriptomics and proteomics of saliva and the oral cavity became increasingly popular subjects of research in recent years, since they represent a non-invasive, safe, and cheap source of complex genetic information. The source of this information-complexity is the large variety of DNAs, RNAs and proteins present in the saliva (Fig. **1**). Salivary DNAs represent (a) the genetic information (genome) of the hosting human body, (b) the oral microbes present in the mouth (oral microbiota), and (c) the infecting DNA-viruses. Salivary mRNAs provide information on the transcription rates of host genes (the human oral transcriptome) and those of the oral microbiota. Other salivary RNAs may indicate RNA-virus infections. Salivary proteins (proteome) represent both genetic information (i.e. the mass-spectrometry reveals amino acid sequences, which help to elucidate the encoding genes) and help to understand the translational regulation of the host body and the oral microbiota. This explains the increasing interest on this subject in several fields including dentistry, medicine and microbiology. 

Many aspects of salivary genomics and proteomics were reviewed in recent years. The diagnostic applications of saliva were covered rather extensively [1-4]. Other reviews highlighted the possible use of saliva in cancer diagnosis [5- 8], in the diagnosis of systemic diseases [9], in microbiota analysis [10] in proteome analysis [11] in psychobiological medicine [12] and in forensic dentistry [13]. However, most of the above reviews discussed a certain specific aspect of the field [5-13]. Other summaries either gave a short general overview [1, 3, 4, 9], or included many other aspects, which are not related to salivary genomics and proteomics (e.g. secretory rates, nonpeptide hormones, ionic changes etc.) [2]. The aim of the present review is to give an integrative overview of salivary genomics, transcriptomics and proteomics including the description of saliva collection, handling and storage; diagnostic possibilities as well as future perspectives.

## SALIVARY GENOMICS, TRANSCRIPTOMICS AND PROTEOMICS: COMPARISON WITH OTHER SAMPLE SOURCES

### Cytologic and Biopsic Collection of Samples

Colpocytology, buccal cell cytology, and several incisial-, needle-, and excisial biopsic sample collection methods (etc.) are usually used for the analysis of local tissues and organs [14-16]. Advantage of such samples is that clear evidence may be reached related to the target tissue [16]. The disadvantage of such sampling is that such methods (especially biopsy methods) are invasive and mostly rather unpleasant for the patients. Another disadvantage is exemplified by their above-mentioned advantage: they provide a local information and are rather unsuitable for the general screening of the organism, although it is true that, they also contain the whole human genome (DNA) [17]. Cytological and biopsy samples are usually sources for detection of local changes related to transcriptome (mRNA) and proteome. Such samples are also used for verifying infections of the target organ by identifying microbial or viral RNA and DNA [16, 18].

### Blood and Body Fluid Sampling

**Fig. (2):**
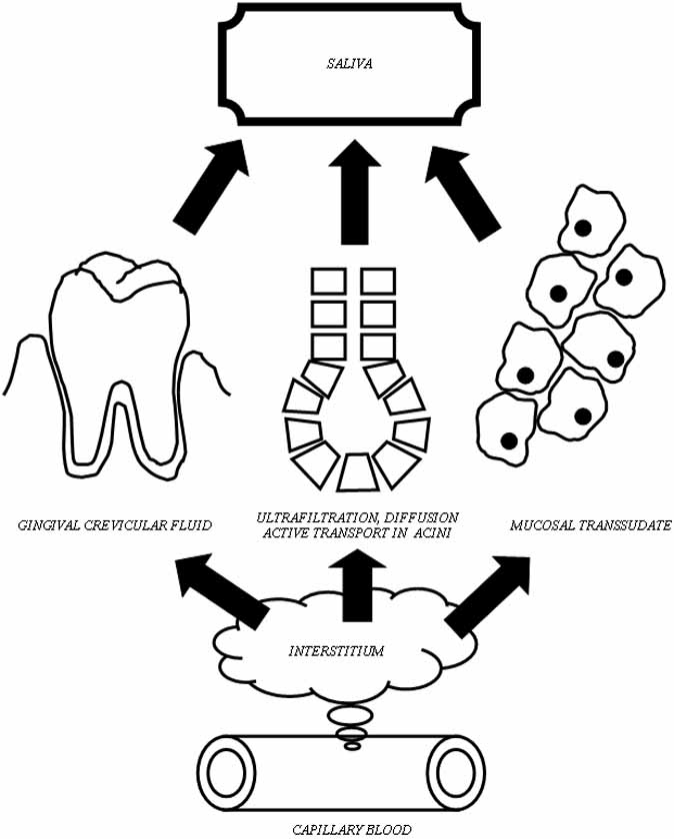
Routes of DNAs, RNAs and proteins of blood origine into the saliva.

Blood and body fluid sampling such as collection of blood and saliva is different. Such samples represent more information related to general conditions of the whole body, which is advantageous for screening purposes [19]. Disadvantage of such sampling is that there is usually no clear evidence related to the origin (localization) of the alterations detected. Blood sampling also has the disadvantage that it is invasive and unpleasant [17]. On the contrary, collection of saliva is noninvasive and not unpleasant at all [17]. *Blood samples* contain many mRNAs and proteins from several body sources, and blood is a source of human genome (human DNA), too. Blood may also contain RNAs and DNAs of infecting viruses and microbes [20]. *Whole saliva* is a source of both the human and oral microbiotal genome. Saliva is also a good tool to detect systemic changes of mRNA and proteome because it is blended with contaminating blood, gingival crevicular fluid, mucosal transudate and also ultrafiltrate of the salivary glands' acini [2] (Fig. **2**). Further, alterations of oral mucosal cells, salivary glands and oral microbiota are also detectable *via *analysis of salivary mRNA and proteome. Finally, saliva contains RNAs and DNAs of infecting microbes and viruses of both local tissues (i.e. oral mucosal cells, salivary glands) and also tissues located in other part of the body [14, 20].

### Comparison of Blood, Oral Mucosal Cell and Saliva Samples

Regarding to *DNA* quality blood samples seem to be superior to all other DNA sources, usually all of the samples could be genotyped, amplified or sequenced [17]. DNA yield from saliva is also rather good in quality, the majority of the samples could be genotyped, amplified or sequenced [17, 21]. Quality of buccal cell DNA seems to be poor [17, 21]. Samples of buccal cells collected with swabs were found not suitable for genotyping, and could be weakly amplified in a comparative study [17]. Buccal cell samples collected with foam-tipped applicators (FTA) could be genotyped in the majority of the cases, however this kind of sample was not suitable for amplification (probably because of the rather small DNA-yield) [17]. The above findings are interesting all the more, because desquamating mucosal cells are the likely source of salivary DNA as well [1]. It might be that DNA of spontaneously desquamated cells - present in saliva - is more intact than that of artificially removed cells. For *mRNA* analysis all sources including blood [22, 23], mucosal cells [24] and saliva [25, 26] can be used effectively. However, there are some differences related to the origin of mRNA present in a certain kind of samples as pointed out above. Regarding to *protein* analysis all the three sources may be used, and the protein-yield is good in all cases. However, it should be considered that there are proteins of different origin present in most of these samples as mentioned above.

## GENOMIC, TRANSCRIPTOMIC AND PROTEOMIC CONSTITUENTS OF SALIVA

### Genome (Human DNA)

The value of total DNA content in human whole saliva was found in a range between 1.8 – 128.4 μg/mL with a mean value of 21.6 μg/mL [17]. In other studies higher values like 40.3 ± 36.5 μg/mL [27] and 77.5 ± 51.5 μg/mL [21] were also reported. A proportion of roughly 70% is of human origin [27] the other 30% is originated from the oral microbiota (and viruses if any). Although it was not jet investigated in detail, it is likely that desquamated oral mucosal cells represent the main human DNA source of saliva. The quality of salivary DNA yield is good, 72% to 96% of samples could be genotyped [17, 27], 84% could be amplified [17] and 67% could be sequenced [17]. 

### Transcriptome (Human mRNA)

Although rRNSs and tRNAs are rather stable in the cell, mRNAs are usually rapidly degraded (which may happen in a few minutes). Partly because of these reasons mRNA constitutes a small proportion of total cellular RNA comparing to the much more abundant rRNA and tRNA fractions. Consequently, extracellular RNAs belong mostly to rRNA and tRNA fractions, whereas the proportion of mRNA believed to be the smallest. It would also be likely that, a high proportion of the salivary RNA is of microbial origin because of the rather rich oral microbiota. Based on the above considerations some authors suppose the absence of detectable amount of *human* mRNAs in a cell free saliva [28]. Although this opinion seems to be a bit exaggerated [29] (see below), but we would like to point out that caution should be used in interpreting saliva-based mRNA expression studies without the technical exclusion of possible DNA contamination artifacts [28, 29].

The total RNA level in *cell free* (centrifuged) whole saliva is ranging from 0.108 ± 0.023 μg/mL [25] to 6.6 ± 3.6 μg/mL [26]. Interestingly, the majority of RNAs present in *cell free* whole saliva is genuine *human mRNA* [29]. The detectable amount of distinct *human* mRNA specii is between 3,000 [25] and 6,400 [26]. These represent roughly 16% [25] and 13% [26] of the included sequences of applied *human* arrays, respectively, and 28% of the protein-coding *human* genes (calculating with middle value of estimations between 20,000 - 25,000 [30, 31]). The proportion of function-unknown salivary mRNSs is about of 27.5% [32]. There are roughly 200 salivary mRNAs found in all the individuals termed "normal salivary transcriptome core" (NSCT) [25, 26, 32, 33]. However, the overlap of individual mRNA stocks are usually higher, 419 and 570 identical transcripts were found in 90% and 80% of the subjects respectively [32]. 

There is also a comparable amount of RNA in isolated parotid saliva (3.6 ± 1.5 μg/mL [26]). However, it contains lower number (4,778 transcripts) of informative mRNAs than whole saliva does [34]. There are also high number of mRNA in sublingual saliva (1,831 transcripts), submandibular saliva (1,543 transcripts), gingival crevicular fluid (2,689 transcripts), and desquamated oral epithelial cells (3,142 transcripts) [34] indicating that, mRNA enters whole saliva from various sources [34]. 

### Microbial DNA and RNA

There proportion of oral microbiotal DNA is roughly 32% indicating a significant amount of non-human genome in whole *not-centrifuged* saliva [17]. Although there is no concrete data available in the literature about the whole amount of non human RNAs in whole *not-centrifuged* saliva, a comparable proportion as in case of DNA is likely. (As mentioned above, the majority of RNAs in *centrifuged* whole saliva is of human origin [29] but in this case microbiota is removed).

### Salivary Proteome

The total amount of proteins in whole saliva is ranging between 0.5 to 3 mg/mL. This proteome consists of roughly 1,000 distinct protein sequences [26], from which around 300 sequences are of human origin [32]. A proportion ranging between 22.8% to 28.7% of detected human sequences have an unknown function [32], which is rather similar to the proportion of mRNAs with no functional identification [32]. The co-existence of a certain protein sequence and its own mRNA is detectable in a rather high proportion of proteins with exact values ranging between 70% to 93%, which is depending on the actual methods used [32]. However, only a little correlation of the change of the amount of a certain protein and the alterations of its mRNA level were found under a particular pathological condition, like the Sjögren's syndrome [26]. Latter finding gives an experimental proof of our general assumption that both protein and mRNA markers are important and provide a non-redundant information [26].

## COLLECTION, HANDLING AND STORAGE OF SALIVA FOR DNA, RNA AND PROTEOME ANALYSIS

### Collection of Saliva

Whole saliva can be collected by drooling into a vial with forward tilted heads, or by allowing the saliva to accumulate in the mouth and than expectorate it into a vial [35, 36]. Isolated parotid saliva may be collected with direct cannulation of the parotid duct, or with the use of parotid cup (a plastic container stabilized on the mucosal surface *via *a pocket enabling negative pressure). Mixed submandibular/sublingual saliva may be collected with direct cannulation of submandibular duct [26].

### Handling of Saliva

Since the main source of salivary DNA is given by desquamated oral mucosal cells [1], preclearing (i.e. centrifuging, microfiltering) of saliva before DNA analysis is not suggested. In contrast, for RNA analysis saliva sample is usually centrifuged (i.e. 2,600 x g, 15 min. 4°C [25, 26]), because the majority of RNAs present in *cell free* (centrifuged) whole saliva are genuine *human mRNA*s [29]. However, the microfiltration of saliva (i.e. instead of centrifuging) is not suggested for RNA analysis, because majority of salivary RNA is macromolecule-associated (see also below) and will not pass through 0.22 μm or 0.45 μm pore size microfilters [34]. For RNA studies focusing on oral microbiota, any kind of preclearing of saliva should be avoided. For analyzing salivary proteome of human origin, saliva may be precleared by centrifugation (i.e. 10,000 x g; 10 min. 4°C) and/or microfiltered by 0.22 μm or 0.45 μm pore size microfilters [35, 36]. However, it should be considered that, high molecular weight macromolecule complexes may be sedimented by centrifugation or blocked in pores of microfilters leading to certain loss of such complexes (and quick obstruction of filters). To avoid such loss of macromolecule complexes a lower g-value centrifugation (i.e. 2,600 x g 15 min. 4°C [25, 26]) may be used, however, in this case microbial contamination of the sample is somewhat higher. In case that, the proteome of oral microbiota is targeted, all kind of preclearing of saliva samples should be avoided.

### DNA Stability

In appropriate buffers of DNA extraction kits saliva can be stored at room temperature for up to at least 1 year [17]. However before adding such buffers saliva should be stored on ice (+4°C) to prevent bacterial (or other microbial growth) and to decrease salivary DNase activity. Saliva can also be frozen and stored on -20°C or -80°C before DNA extraction [37]. Extracted DNA can be also frozen and stored on -20°C or -80°C until use [17]. 

### RNA Stability 

There are several microbial and/or human RNases present in saliva which may destroy RNA molecules, in some cases even with a half-lives of a few minutes (range between 0.4 to 12.2 min.) [34]. Interestingly human salivary mRNAs are more stable in saliva than exogenous ones (i.e. mouse mRNA added to saliva [34]) indicating that human mRNA is protected by certain human-specific mechanisms against salivary nucleases [34]. This protective phenomenon likely occurs because of RNA-macromolecule interactions [34] and can be abolished with detergents [34]. (The phenomenon that RNAs associate with other macromolecules is known also in serum and plasma. Such complexes are called *particle-associated RNAs* [23]). Possible associated proteins of salivary mRNA may be apoptotic bodies of desquamated mucosal cells [22, 34] and several saliva proteins like mucines [34]. It may also be hypothesized that, salivary chaperone Hsp70 [35, 38] may take part in the protection of human mRNA, too. Because of premised possible role of proteins in salivary mRNA stability, addition of protease inhibitors to salivary samples can be useful even in case of mRNA analysis [25, 26]. Cooling of saliva samples on ice (+4°C) is also suggested [25, 26]. 

Because of above mechanisms, most of salivary RNAs are partially degraded only [29, 34]. On the average salivary mRNA represents 42% of its respective (original) full length [29]. In contrast to blood the degradation pattern is rather random with only a slightly more degradation at the 5' end (if any) [29, 34]. Further, those of long transcripts have an increased chance of being degraded than shorter [29, 34]. These findings indicate a predominant endonuclease activity in saliva [29, 34] in contrast to blood, in which the activity of exonucleases is typical [19]. Because of above, use of RNase inhibitors (especially against endonucleases) is recommended to avoid destruction of salivary RNAs. Although with the use of certain RNase inhibitors mRNA can be stabilized for longer run even in room temperature [39], however cooling of saliva samples on ice (+4°C) is still useful [26]. RNA can be preserved in saliva also by freezing at -80°C [25], in this case supplementation with stabilizing "RNAlater" reagents may be suggested [26]. 

### Protein Stability

Precleared saliva can be stored on ice (+4°C) without significant protein degradation only for few hours, and without preclearing the degradation is even quicker. Consequently addition of protease inhibitors is advantageous especially for time consuming analysis procedures. Freezing frequently resulted in significant protein precipitation, even if quick freezing is used, and the frozen sample can be stored for a few days only without any further damage at -20°C. Somewhat longer storage is possible in liquid nitrogen or at -80°C, however proteins are not stable for a longer run even in such conditions. 

## SYSTEMS BIOLOGY OF SALIVARY MICROBIOTA

### Complexity of the Oral Microbiota

The microbiota of the oral cavity consists of more than 600 microbial specii (or not yet cultivated phylotypes) including predominantly bacteria, but also fungi and protozoa [40-43], from which 347 clones are predominant in subgingival pocket [43], others are more frequent in the supragingival bacterial biofilm (i.e. dental plaque). However in individual hosts just a subset of all specii can be found [44], indicating a high degree of interpersonal variability. The subgingival bacterial community in a single individual was found to consist of 25 to 34 [45], 59 [46] or 72 to 99 [43] specii and phylotypes in different studies. Similarly, supragingival tooth-surface attached biofilm (dental plaque) consisted of 11 to 29 [47], or 32 [48], or 52 [49], or up to 94 to 114 (pooled sample) [50] specii and phylotypes in several studies. 

### Microbiota of the Saliva

Despite the important antimicrobial activity of saliva [38, 51, 52] the complexity of salivary microbiota was similar to the oral microbiota above including 37 [40] or 33 to 53 [44] different bacterial specii and phylotypes and 1 to 3 yeast (*Candida*) [53] specii, (altogether up to 56 specii and phylotypes). It is likely that, the total number of salivary specii is even higher [44]. To understand the high number of specii despite the antimicrobial saliva activity it should be considered that, the bacterial metabolism alters when certain bacteria get in contact with saliva [54], and several bacteria can even ferment and grow on saliva [55]. Further, microorganisms attached to the surfaces of the mouth and teeth are continuously shed into the salivary fluid, and bacteria residing in the periodontal pockets are constantly washed into saliva by the gingival crevicular fluid [44]. Because of the above mentioned constant microbial replacement, salivary microbiota reflect to many changes occurring in any part of the mouth as a "fingerprint" of the whole oral microbiota [44, 56].

### Effect of Salivary Microbiota on other Parts of Gastrointestinal Tract

Increased expression of molecular chaperones (especially DnaK) strongly increases the acid tolerance of salivary bacteria (i.e. *Streptococcus mutans*) on pH 5 (or even lower) [57]. Such a low pH occur regularly in the deeper area of tooth surfaces attached bacterial biofilm (i.e. dental plaque) [57] indicating that, many of bacteria present in such biofilm may survive in rather low pH. Thus, such bacteria also may survive passing through the stomach. This route opens the possibility that, oral microbiota may have an influence on and alter the microbiota of other parts of gastrointestinal tract. For example, *Helicobacter pylori* bacteria, (a known cause of peptic ulcer disease and chronic gastritis) was found in higher prevalence in saliva than in feces, indicating that oral rout (including oral-oral route like kissing) may be an important means of transmission of this infection [58]. Some *Lactobacillus* specii present in the faeces were also found to be transient (allochthonous) to the intestine and originate from the oral cavity [59]. It is likely that, recently developed oral microbiota diagnostic systems based on 16S rRNA microarrays [10, 60] may lead to recognition of numerous similar interference of oral and gastrointestinal microbiota. Although the microbiota normally configure to match the usual normal community structure, in some cases as-yet uncharacterized properties may alter the microbial balance [61, 62]. It may not be excluded that, certain transient species of oral origin can be responsible for such alterations, leading to several pathologic conditions of the gastrointestinal tract. 

## SALIVARY DNA, RNA AND PROTEOME IN THE DIAGNOSIS OF DISEASES

### Early Cancer Diagnosis

In case of saliva two major facets of cancer diagnosis should be distinguished, such as diagnosis of oral cancers (getting direct contact with saliva) and of those in other locations. Advanced stage *oral cancers* are usually detectable by inspection of the oral cavity. In contrast, early stage oral carcinomas are not viewable, frequently not diagnosed or treated in time, because even microscopic level for a progressive cancer can be too late for successful intervention [6, 63]. Similarly, tumors of *other locations* are also usually not diagnosed early enough, because they are out of sight in early stages and also because they are frequently not viewable even in advanced stages (depending on location). Since the prognosis of advanced stage cancers are much worse than of early stage ones, highly sensitive methods for both oral and other located early cancer detection are needed to reduce lethal outcome of these dangerous diseases [63]. 

There are some tumor specific *DNA markers* in serum and other body fluids may be used for the diagnosis of both *oral *and* other tumors*, based on the assumption that the initiation and progression of malignant tumors is driven by the accumulation of specific genetic alterations [1, 6]. In saliva, mutated salivary DNA at p53 gene was found in 62.5% of oral cancer patients [64]. Similarly, an increased content of mitochondrial DNA was found in the saliva (also in tumor tissue [65]) of head and neck cancer patients [66], which decreased after the surgical removal of such tumors [67]. Detection of HPV (human papilloma virus) DNA in saliva also enables the detection of HPV-related head and neck cancers [68]. Similarly, detection of DNA (or proviral DNA) of other tumor-inducing viruses like HIV (human immunodeficiency virus) [69] and HHV-8 (human herpesvirus 8) [70] in saliva may also be useful for oral and/or other located cancer risk assessment. 

Analysis of serum* mRNAs* can also be a good and sensitive tool for detection of certain oral and other tumors [71-73]. Especially global mRNA-profiling of serum seems to be promising [71]. However collection of blood is unpleasant for many of subjects [17], it is invasive and expensive to some extent. Consequently, the collection of saliva seems to be more suitable for the screening of larger populations. Profiling of *salivary mRNA* indicated four major biomarkers of oral cancer including interleukin-1-β (IL1β), interleukin-8 (IL8), ornithine decarboxylase antizyme-1 (OAZ1) and spermidine/spermine N1-acetyltransferase (SAT). Regression tree (CART) analysis of these four, elevated biomarkers was able to distinguish patients with T1 and T2 oral cancer from control subjects with very high sensitivity (91%) and specificity (91%) [63]. Beside the above markers, detection of tumorigenic virus RNAs, like HIV-1 may also be useful in evaluating risk of malignancies [20].

Salivary *proteome* can also be used for tumor detection. Increased salivary level of defensin-1 [74], cancer antigen CA15-3 [5, 75], tumor marker proteins, like c-erbB-2 [5, 75] or CA-125 [76] and antibodies against the tumor suppressor protein, p53 [77] seem to be promising markers for both oral and other malignancies [2]. A future global proteome profiling of saliva with newly developed methods of proteome analysis [78-82] would likely resulted in further candidate peptide sequences of sensitive tumor detection [3]. 

### Other Systemic Conditions

There are significant changes of salivary proteome and transcriptome also in the case of *Sjögren's syndrome* [26]. A recent study indicated 16 down-regulated and 25 upregulated distinct amino-acid sequences in whole saliva proteome, from which 10 up-regulated and 6 down-regulated proteins seemed to be statistically significant biomarkers of Sjögren's syndrome [26]. This study also indicated a higher level of total RNA of both whole and parotid cell free saliva [26]. In whole saliva 162 mRNAs were up-regulated (at least 2-fold), whereas only few were down-regulated. 27 mRNAs were up-regulated at least 3-fold, and 13 mRNAs were upregulated more than 10-fold [26]. From the 27 (at least 3-fold upregulated) genes 19 were interferon-inducible, or were related to lymphocyte infiltration, and antigen presentation known to be involved in the pathogenesis of Sjögren's syndrome. The mRNA of an interferon-α inducible protein (G1P2) was 500-fold upregulated [26]. Taking together these data, it is obvious that the analysis of salivary proteome and transcriptome (especially, when used together) provides a great possibility for both the better understanding and the improved diagnostics of Sjögren's syndrome [26] and other diseases.

Psychological *stress conditions* also induce significant changes in both salivary proteome and transcriptome. The increase of salivary amylase is a known proteomic indicator of psychological stress [36, 83] and sympathetic activation [84, 85]. A recent paper indicated that amylase specific salivary mRNA level also increases significantly after stress with some delay [86]. Besides the above stress-markers, decrease of secretory IgA [87], and increase [36] of immune-modulator defense protein salivary chaperone Hsp70 [35, 38] was also reported. Global profiling of genome, transcriptome and proteome present in saliva likely lead to the recognition of numerous other stress-markers and stress sensitivity markers in the next future.

A number of other disorders exist, where salivary changes with significant diagnostic value were also characterized. In *Cystic fibrosis* the excretion of an unusual, less effective form [88] of salivary epidermal growth factor (EGF [89]) was reported. There are also characteristic salivary changes in case of *graft-versus-host disease* including the elevated concentration of total protein, albumin, EGF, IgG, and a decreased amount of IgA and IgM in the saliva [90]. In *coeliac disease* the elevated salivary level of IgA antigliadin antibodies (AGA) may be used for screening purposes with moderate sensitivity (60%) and high specificity (93%) [91]. In case of *peptic ulcers* and *chronic gastritis*, *Helicobacter pylori* infection (a frequent cause) can be detected with 84% sensitivity and 82% specificity *via *detection of bacterial DNA in saliva [58]. *H. Pylori* may also be detected with salivary antibodies against this bacterium (sensitivity 85%, specificity 55%) [92]. *Neurocistercosis* can also be identified with the detection of antibodies against *Taenia solium *larvae with a sensitivity of roughly 70% [93]. The above mentioned disorders and molecular markers may also be taken as examples of the next future possibilities opening to recognize sensitive diagnostic markers of further disorders [1, 3, 4, 79]. 

### Virus Infections

Saliva analysis is a good tool for detection of virus infections, based on identification of viral DNAs and RNAs, antibodies against viruses, and viral antigens [2, 94]. DNA and RNA analysis shows moderate to high sensitivity, however a progress related to the accuracy of such methods is very likely in the next future. Proteomic methods like detection of antibodies against viruses and detection of virus antigens are highly sensitive and specific methods available already today (see below). Detection of salivary *viral DNA* is a possible diagnostic tool for screening with a moderate to high sensitivity in case of several viruses like cytomegalovirus (sensitivity 36% [14]), human herpes virus (HHV) type 6 (sensitivity 80% [95]), HHV type 7 (sensitivity 100% [95]), HHV type 8 (sensitivity 30% to 66% [18, 70]) and transfusion transmitted (TTV [96]) virus (sensitivity 39% [97]). Detection of salivary *proviral DNA* (host cell integrated viral sequences) may also be used for such purposes with moderate sensitivity (40% [98]) in case of human immunodeficiency virus type 1 [16, 69, 98]. Detection of *viral RNA *in saliva may also be used with moderate to high sensitivity in detection of viruses like hepatitis G virus (sensitivity 35% to 100% [97, 99]) and HIV-1 (sensitivity 37% to 100% [14, 20, 98, 100, 101]). In the *proteomic level* numerous viruses can be detected with high sensitivity and specificity *via *detection of salivary *antibodies against viruses*. There are highly sensitive methods available for dengue virus (sensitivity 92%, specificity 100% [102]), hepatitis A (sensitivity 99%, specificity 99% [103]), hepatitis B (sensitivity 100%, specificity 100% [104]), hepatitis C (sensitivity 100%, specificity 100%) [104, 105], HIV-1 (sensitivity 95% to 100%, specificity 95% to100% [106-109]), measles (sensitivity 97%, specificity 100% [110]), mumps (sensitivity 94%, specificity 94% [110]), parvovirus type B19 (sensitivity 100%, specificity 95% [111]), and rubella (sensitivity 98%, specificity 98% [110]). Detection of *virus antigens* in saliva is also a possible highly sensitive and specific method in detection of hepatitis B virus (sensitivity 92%, specificity 87% [112]).

### Dental Caries

There are some more or less specific changes of salivary proteome, which may be used for recognizing caries-risk patients. These include decreased level of proline-rich proteins (PRP1 and PRP3), histatin 1 and statherin [113]. Increased numbers of *Streptococcus mutans* and *Lactobacilli* in saliva were also associated with increased caries prevalence [114, 115], and with the presence of root caries [116]. Decreased complexity (fewer number of specii and phylotypes) of the oral microbiota was also found to be a risk for early childhood caries [50]. Although caries related microbiotal data were recognized mainly by analysis of tooth-surface biofilm (dental plaque) but the complex microbiota present in saliva [40] likely reflect to this differences as well [44, 56]. Investigation of salivary microbiota with high capacity automatized nucleic acid extraction and analysis [117] and also global genome, transcriptome and proteome profiling of saliva will likely lead to further new caries related predictive markers in the future.

### Periodontal Inflammation

The presence of certain periodontopathogen bacterial species in saliva reflect their presence in dental plaque and periodontal pockets [45, 56, 118, 119] indicating that saliva may be a good tool to detect bacterial risk factors by salivary DNA analysis of oral microbiota [45]. Such DNA-based methods open new perspectives in both the understanding and the diagnosis of periodontal disorders, since they allow the analysis of non cultivable (yet-to-be cultured) microorganisms as well [45]. Besides genomic methods, proteomic analysis may also become important in the next future. It is known for example that the level of alpha-2-macroglobulin, alpha-1-antitripsin, elastase and also albumin in saliva may be good indicators of gingivitis and/or periodontitis. The level of salivary defense proteins like immunoglobulin, molecular chaperone Hsp70, cystatin S, salivary amylase, calprotectin, hystatins, lysozyme, lactoferrine, defensins, peroxidases, prolin-rich proteins and mucins [38, 51, 52] may also have prognostic value related to the possible passing of gingivitis to periodontitis (where the latter is a more severe inflammation with irreversible destruction) [38, 120, 121]. It is also likely that the global profiling of salivary genome, transcriptome and proteome will lead to recognition of further highly sensitive diagnostic markers of periodontal conditions.

### Oral Candidiasis 

Saliva may also be used for detection of oral fungi [53, 122]. In case of oral candidiasis, salivary fungal counts may also reflect mucosal colonization [123]. Alteration of salivary proteome related to proteins showing antifungal properties like immunoglobulin, calprotectine, histatin-5, mucins peroxidases, basic prolin-rich proteins, molecular chaperone Hsp70 etc. [38, 51, 52] may also have important diagnostic/prognostic value especially in recurrent cases.

## CONCLUSIONS AND PERSPECTIVES

With advances in genomics, transcriptomics and proteomics of saliva, salivary testing in clinical and research settings is rapidly proving to be a practical and reliable means of recognizing several systemic and oral conditions [2, 9]. However, further detailed studies establishing the diagnostic value of saliva in comparison with that of other biomedia, (especially with blood) will be necessary to assess the detailed prognostic and diagnostic value of saliva [4]. At this stage of knowledge saliva seems to be a highly important possible tool for regular screening of larger populations. However, it may also turn out in many cases that saliva is as accurate (or even better) as blood in establishing a definitive diagnosis of certain disorders and monitoring disease progression [1, 4]. However, the road to practical and effective regular use of salivary diagnostics is expected to be promising, but long [6, 7]. For the regular clinical use the analysis should be highly automatized, and coupled with microfluidic technology, enabling a small sample size to be used, avoiding reagents' and waste's cost and allowing that types of assays that are impossible at the macroscopic level [10]. The identification of biomarkers with a proper and definite sensitivity and specificity to as many disorders and conditions as possible is also a prerequisite [7]. Thus, further technological advancement and identification of robust and discriminatory sets of salivary biomarkers is necessary [7] to fulfill all requirements for being regular diagnostic tool for the everyday clinical practice. Despite limitations we experience today, the use of saliva for diagnostic purposes becomes increasingly popular, and as a result, more and more diagnostic tests become commercially available, and are currently used by clinicians and researchers [2]. Taking together all these aspects, it can be concluded that there are rich possibilities in saliva-diagnostics already at present, and the immediate future of this area is even more promising.
